# Anterior pituitary function in critical illness

**DOI:** 10.1530/EC-19-0318

**Published:** 2019-07-24

**Authors:** Arno Téblick, Lies Langouche, Greet Van den Berghe

**Affiliations:** 1 Clinical Division and Laboratory of Intensive Care Medicine, Department of Cellular and Molecular Medicine, KU Leuven, Leuven, Belgium

**Keywords:** critical illness, pituitary function, neuroendocrine axis, CIRCI, NTI

## Abstract

Critical illness is hallmarked by major changes in all hypothalamic–pituitary–peripheral hormonal axes. Extensive animal and human studies have identified a biphasic pattern in circulating pituitary and peripheral hormone levels throughout critical illness by analogy with the fasting state. In the acute phase of critical illness, following a deleterious event, rapid neuroendocrine changes try to direct the human body toward a catabolic state to ensure provision of elementary energy sources, whereas costly anabolic processes are postponed. Thanks to new technologies and improvements in critical care, the majority of patients survive the acute insult and recover within a week. However, an important part of patients admitted to the ICU fail to recover sufficiently, and a prolonged phase of critical illness sets in. This prolonged phase of critical illness is characterized by a uniform suppression of the hypothalamic–pituitary–peripheral hormonal axes. Whereas the alterations in hormonal levels during the first hours and days after the onset of critical illness are evolutionary selected and are likely beneficial for survival, endocrine changes in prolonged critically ill patients could be harmful and may hamper recovery. Most studies investigating the substitution of peripheral hormones or strategies to overcome resistance to anabolic stimuli failed to show benefit for morbidity and mortality. Research on treatment with selected and combined hypothalamic hormones has shown promising results. Well-controlled RCTs to corroborate these findings are needed.

## Introduction

Critical illness is defined as the presence of acute, life-threatening organ dysfunction requiring vital organ support and can be evoked by major trauma, extensive surgery, large-scale burn injuries and severe medical diseases. A hallmark of critical illness, is the immediate initiation of multiple physiologic processes in an attempt to rebalance the complex dynamic equilibrium, commonly known as homeostasis. This so-called ‘stress response’ comprises many tightly controlled neural and endocrine adaptations to provide sufficient energy and hemodynamic stability to survive and overcome the immediate phase after onset of critical illness. Supported by advancements in modern health care such as mechanical ventilation, renal replacement therapy or broad-spectrum antibiotics, the majority of critically ill patients will survive the acute phase of their illness. However, a significant number of patients admitted to the ICU fail to recover sufficiently within a few days and enter a more prolonged phase of critical illness, also known as ‘chronic critical illness’ (1). Although timing of this transition is unclear, after approximately 10 days of critical illness, the severity of illness upon admission is no longer predictive for mortality (2). Depending on the used criteria, 5–30% of the patients admitted to an ICU will eventually suffer from chronic critical illness.

Independent of the underlying cause for admission to the ICU, the hormonal stress response to critical illness follows a biphasic pattern related to the time course of critical illness (3, 4). A first phase, further referred to as the acute phase of critical illness, starts within minutes or hours after the occurrence of the deleterious event. An evolutionary hormonal ‘fight or flight’ state is activated by the abundancy of released proinflammatory cytokines, the overwhelming activation of sensory neurons, the release of catecholamines and/or the presence of pathogens in the bloodstream (5, 6). Further augmented by an illness-induced reduction in nutritional intake, hormonal changes during the acute phase of critical illness are directing the organism toward a catabolic state in an attempt to provide sufficient energy to overcome and survive the insult. A reduction in cellular oxygen and/or energy delivery, and insufficiently activated defense mechanisms induce mitochondrial dysfunction and consequently, a downregulation of cellular metabolism (7, 8). Although survival from previously lethal conditions is enhanced by the provided critical care, recovery does not always follow fluently. When patients outlive the acute phase of critical illness but remain dependent on vital organ support, sometimes for multiple weeks, the central activation of most neuroendocrine axes is attenuated, together with complex alterations in peripheral hormone levels.

Two tandem key players in regulating the stress response in critically ill patients are the hypothalamus and pituitary. The hypothalamus, the major control center of the different neuroendocrine axes, gains a complex set of sensory input from a variety of internal and external stimuli. This collection of information together with the input of endocrine feedback loops triggers the hypothalamus to produce and secrete tropic hormones in the hypophyseal portal system mainly targeting the anterior pituitary. Highly regulated by these stimulating or inhibiting hypophysiotropic hormones and various feedback loops, the anterior pituitary produces a set of hormones targeting peripheral glands, such as the thyroid, the adrenal or the gonads or end organs directly such as the liver, muscle and bone.

In this paper, we will review the anterior pituitary function and the five main neuroendocrine axes during health and critical illness, both in the acute and the chronic phase.

## The five main neuroendocrine axes

### The somatotropic axis during health

**Figure 1:**
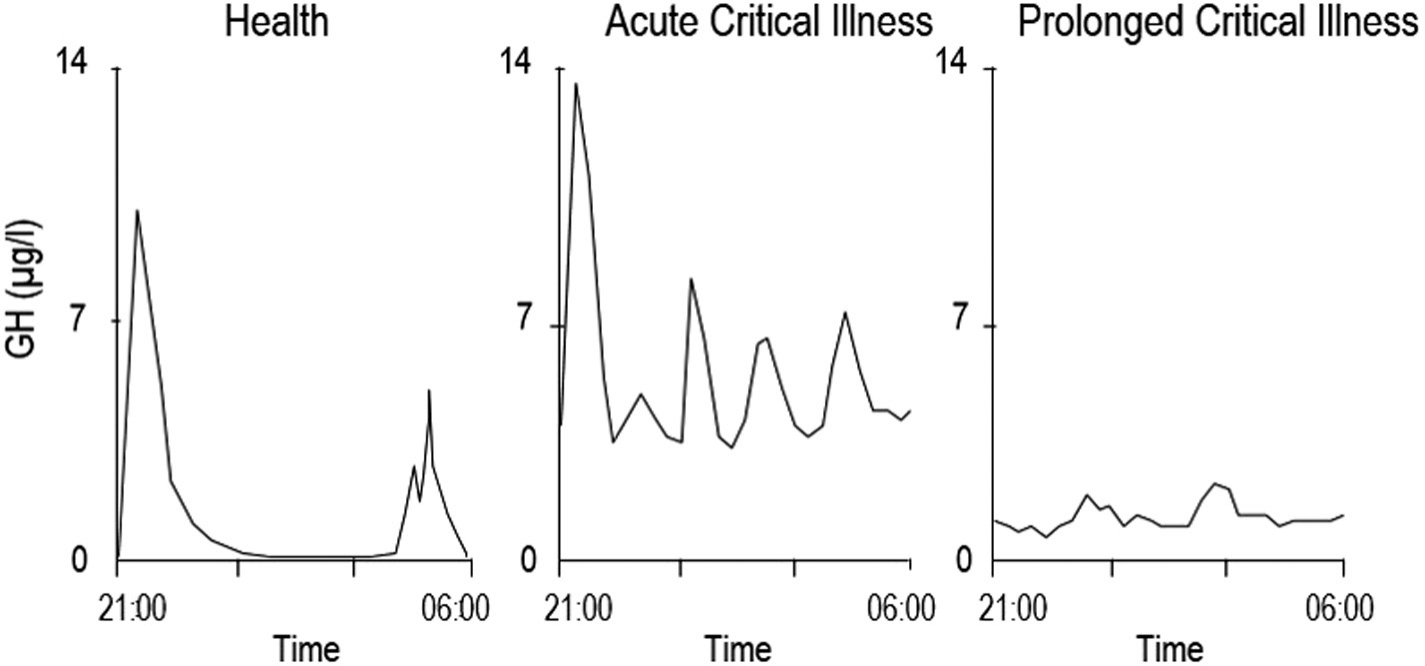
Changes in GH during critical illness. During the acute phase of critical illness, the nocturnal secretion of growth hormone is augmented with an increase in pulse amplitude and pulse frequency. In prolonged critical illness this pulsatile pattern becomes blunted. Adapted, with permission, from (3).

Growth hormone (GH), the most abundant pituitary hormone, is synthesized by the somatotropes in the anterior pituitary. Hypothalamic GHRH stimulates production and release of stored GH into the bloodstream. Counteracting, somatostatin inhibits production of both GHRH and GH. Upon stimulation, GH is secreted in a pulsatile manner, noticeable by the highly fluctuating serum concentrations, with peaks every 3–4 h followed by a decline down to undetectable levels (Fig. 1). Whereas GHRH levels positively correlate with the amplitude of the GH peak, high somatostatin levels determine the end of the GH release and thus the initiation of the interpulse-trough (9). On top of this straightforward interplay between two regulating hormones, a third key player in GH production is the in the stomach synthesized hormone ghrelin. Through binding with the GH secretagogue receptor (GHS-R) at the level of the pituitary and hypothalamus, ghrelin stimulates, directly and indirectly, pituitary secretion of GH. Ghrelin has similar but less potent effects on pituitary ACTH and PRL secretion (10). Furthermore, ghrelin has a profound orexigenic effect, which appears to be mediated by the GHS-R but in a GH-independent fashion, as suggested by GH-deficient animal studies (11). GH, acting in a direct and indirect manner, the latter by stimulating the hepatic production of the para-/autocrine hormone insulin-like growth factor-I (IGF-I), is named for one of its main functions: mediating linear bone and organ growth. Whereas adequate nutritional intake and adequate sex hormone levels are the main growth promoters during respectively infancy and puberty, GH takes up this role during childhood. In adults, GH remains important as a regulator of metabolism. The set of complex actions of GH and IGF-I on carbohydrate, fat and protein metabolism is highly variable depending on the nutritional status of the individual. In well-nourished, healthy individuals IGF-I stimulates protein synthesis and peripheral free fatty acid uptake, whereas in prolonged fasting, GH will enhance the release and oxidation of free fatty acids and antagonize insulin actions.

### The somatotropic axis during critical illness

GH serum concentrations start to rise in the hours after the onset of critical illness. An increment in both amplitude and frequency of GH peaks and the loss of the typical troughs during the interpulse periods contribute to the elevated serum concentrations (Fig. 1) (4, 12, 13). Furthermore, the hepatic GH receptor function is oppressed, often referred to as ‘peripheral GH resistance’, leading to low circulating levels of IGF-I, IGF-binding protein 3 (IGFBP-3), its acid label subunit (ALS) and GH-binding protein (GHBP) (14). Combined, these alterations in the GH axis lead to a shift from the anabolic effects of IGF-I to more catabolic actions of GH, such as lipolysis, insulin antagonism and to immune stimulation (15). When recovery does not ensue within a week and patients enter the chronic phase of critical illness, the pulsatile pattern of GH secretion fades and GH peaks become blunted with IGF-I, IGFBP-3 and ALS levels remaining low (Fig. 1). Interpulse GH concentrations also decrease but still appear to be higher than in healthy subjects (16). Whereas in the acute phase of critical illness hepatic GH resistance has a key role in altering the GH axis, the main driver of hyposomatotropism during the prolonged phase of critical illness is thought to be an impaired hypothalamic drive. Hepatic GH resistance does not seem to persist during chronic critical illness (17). This hypothesis is supported by a high GH responsiveness to administration of GH secretagogues (GHRPs) in chronic critically ill patients. Indeed, restoration of pulsatile GH secretion pattern can be evoked by the administration of GHRP, alone or with the co-administration of GHRH, leading to a six-fold and ten-fold increase in amplitudes of GH serum peaks, respectively. Strikingly, administration of GHRH alone is not capable of restoring the typical pulsatile pattern of GH secretion (18). Besides an altered hypothalamic drive, another possible contributor to the attenuated GH levels during chronic critical illness may be the scarcity of the active form of ghrelin, the endogenous ligand of the GHS receptor and a powerful GH secretagogue (19). The low circulating IGF-I and its binding proteins levels are associated with biochemical markers of impaired anabolism, such as low serum osteocalcin and leptin (20). The chronic GH deficiency, with reduced anabolism and ongoing catabolism, thus likely contributes to the wasting syndrome, a hallmark of chronic critical illness.

A large RCT, investigating the effect of high-dose GH injection to prolonged critically ill patients, unexpectedly marked a doubling in mortality in the intervention cohort (21). Since GH resistance at least partially resolves in the chronic phase, it is likely that such high doses of GH, and consequently high levels of IGF-I, evoked toxic side effects such as excessive fluid retention, hypercalcemia and pronounced insulin resistance with hyperglycemia. Although small studies showed the ability of GHRP-2 to restore a normal GH pulsatile pattern in severe ill patients, and of the combination of GHRP-2 and thyrotropin-releasing hormone (TRH) to induce an anabolism and suppress catabolism in prolonged critically ill patients (16, 22), the clinical outcome of infusion with GH secretagogues has not yet been studied. Also substitution with ghrelin has recently been investigated in smaller animal and *in vitro* studies and appeared to enhance autophagy, reduce catabolism and improve hemodynamics (23). As ghrelin induces appetite, infusion of ghrelin during the chronic phase of critical illness when patients restart oral intake may enhance food intake and could lead to improvement in clinical outcome (24). Large-scale RCTs in humans, to corroborate these findings, have not yet been performed.

### The thyroid axis during health

Stimulated by the hypothalamic TRH, thyrotropes in the pituitary gland produce and secrete thyroid-stimulating hormone (TSH) in a dual fashion: a basal secretion with a circadian pattern (nocturnal levels up to a twofold of daytime levels) and pulses approximately every 90 min (25). TSH binds the G-protein-coupled TSH receptor (TSH-R), predominantly but not exclusively found on thyroidal cells, adipocytes and orbital fibroblasts. Activation of the thyroidal TSH-R induces thyroid gland growth, transformation of cell morphology, iodine metabolism and synthesis of thyroid hormones thyroxin (T4) and to a lesser extent triiodothyronine (T3). In peripheral tissues, T4 crosses the cell membrane through specific transporters and subsequently undergoes outer- or inner-ring deiodination, resulting in the formation of respectively the metabolic active T3 or the metabolic inactive reverse T3 (rT3). The nuclear thyroid hormone receptor (THR) exists in three active isoforms (TRα1 and TRβ1 and 2) and one inactive isoform TRα2. As the THRs are found in virtually every human organ, physiologic effects of thyroid hormones are heterogeneous, ranging from basal cellular metabolism to growth and facilitating local tissue functions. Thyroid hormones T4 and T3 exert inhibitory feedback control on both the hypothalamus and pituitary.

### The thyroid axis during critical illness

**Figure 2:**
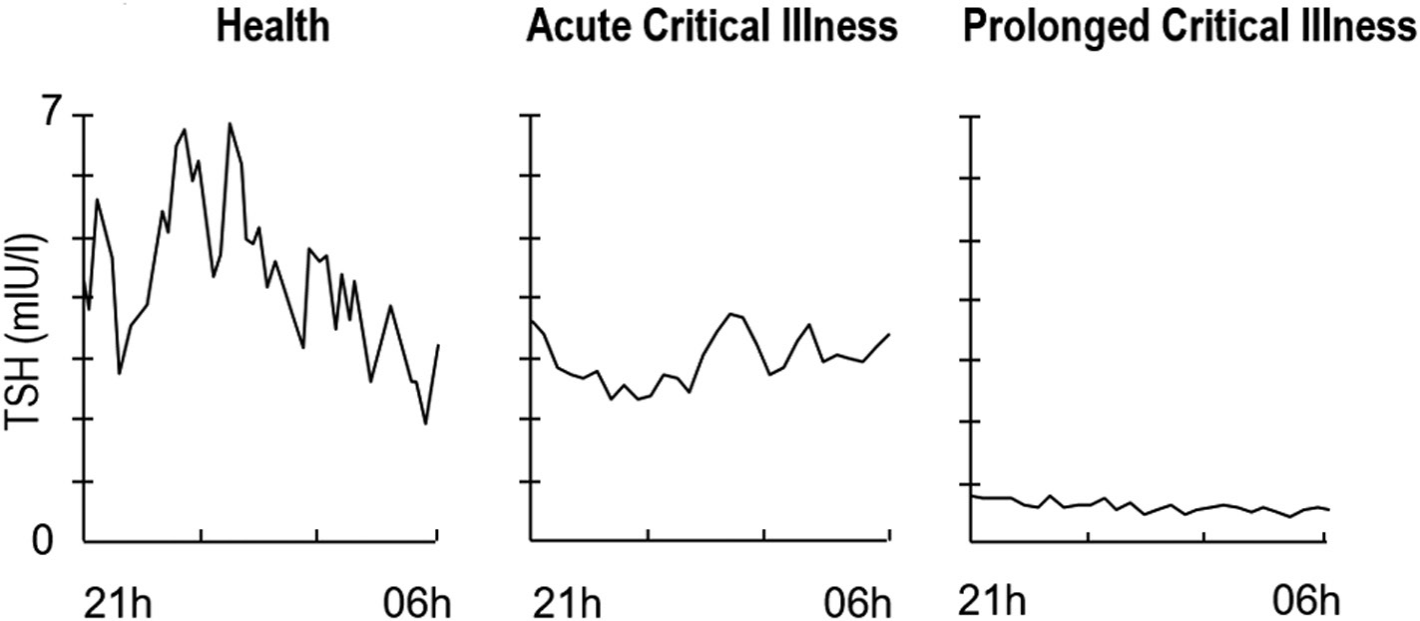
Changes in TSH during critical illness. The typical nocturnal surge of TSH disappears during the entire course of critical illness, mean TSH levels in the acute phase are not dramatically altered. Conversely, TSH levels are distinct lowered during the chronic phase of critical illness. Adapted, with permission, from (3).

Within hours after the onset of critical illness, circulating levels of T3 rapidly decline, whereas rT3 plasma concentrations increase. These characteristic changes are due to an altered peripheral conversion of T4 with a typical decrease in the peripheral activity of the activating type 1 deiodinase (D1) and increase in the inactivating type 3 deiodinase (D3) (26, 27). Apart from the absence of the nocturnal surge in TSH and a swift and transient rise in TSH and T4, both hormone levels remain relatively normal (Fig. 2) (28). These changes are often referred to as ‘non-thyroidal illness’ (NTI). Alterations in the affinity of thyroid hormone-binding proteins, thyroid hormone transporters and the nuclear THR further contribute to the NTI.

Low circulating levels of T3 reduce energy expenditure, but also to optimize bacterial killing capacity through increased D3 activity in cells of the innate immune system, which could play a role in the observed reduction in nosocomial infections and therefore could be beneficial, at least in the acute phase of critical illness (29, 30, 31, 32). When patients remain dependent on vital organ support for multiple days or even weeks and are under full nutritional support, by enteral and/or parenteral feeding, TSH and T4 levels start to decline with T3 levels remaining low. Similar to the observed alterations of the GH axis during prolonged critical illness, the pulsatile pattern of TSH fades and secretory peaks become blunted (Fig. 2) (33). Interestingly, in chronic critical illness, peripheral tissues seem to adapt to the sustained low T3 levels by enhancing local hormone availability and effectiveness. Indeed, a peripherally increased expression of the thyroid membrane transporter MCT-8, upregulated D2 expression and increased TRα1/TRα2 ratio expression have been observed (34, 35). Nevertheless, ongoing low levels of T3 have been associated with more pronounced catabolism and worse outcome (18, 36). Furthermore, ICU patients who received an infusion of TRH combined with GHRP-2 showed normalized thyroid hormone levels and lowered markers of hypercatabolism (18). During the acute phase of critically ill patients, especially with the concomitantly reduced nutritional intake, treatment of the low T3 levels, in the absence of preadmission thyroid pathology, is probably not indicated (37). Whether or not the central hypothyroidism during the chronic phase of critical illness would benefit from treatment is not yet clear from available small human studies (38). In animal models, normal substitution doses had no impact on thyroid hormone levels, due to the highly increased metabolism. High doses of T4, T3 or the combination could restore normal hormone serum concentrations but led to overtreatment with further suppression of TSH and rise in rT3 (39, 40, 41). Interestingly, TRH infusion alone led to a twofold increase in basal TSH secretion, and co-infusion of TRH and GHRP-2 increased pulsatile TRH secretion by five-fold in prolonged critically ill patients (18). In addition, also anabolic markers (osteocalcin and leptin) appeared to be higher compared to placebo-infused controls (20). In contrast to T3 and T4 infusions, treatment with a hypothalamic releasing factor allows normal feedback inhibition, but until today RCT’s testing this treatment on short- and long-term clinical outcome of prolonged critically ill patients are lacking.

### The adrenal axis during health

Pro-opiomelanocortin (POMC), synthesized in the corticotropic cells of the pituitary, is spliced to adrenocorticotropic hormone (ACTH) by proteolytic cleavage enzymes. ACTH is stored into dense core secretory granules and released into systemic circulation either spontaneously, in pulses every 30–40 min with a diurnal rhythm or acutely, upon stimulation with hypothalamic CRH (42). CRH activity is potentiated by the presence of vasopressin (VP). At the adrenal gland, ACTH binds to the G-protein-coupled melanocortin-2 receptor (MC2R). MC2R activation leads to increased expression of cholesterol uptake receptors (such as low-density lipoprotein-receptor) and the cholesterol synthesis enzyme 3-hydroxl-3-methyl-glutaryl-coenzym A reductase (HMG CoA reductase). Unesterified intracellular cholesterol is then converted to prenenolone by P450 side chain cleavage enzyme. The expression of this enzyme, that is, the rate-limiting step in adrenal steroidogenesis, is also upregulated by ACTH-induced MC2R activation (43). Likewise, the final enzyme for cortisol synthesis, 11-β-hydroxylase, which converts 11-deoxycortisol into cortisol is upregulated upon ACTH stimulation. In contrast to pituitary hormones, steroid hormones are not stored in the adrenal gland but directly secreted after synthesis. This at least partially explains the tight correlation between serum ACTH and serum cortisol concentrations during health. Cortisol mitigates its own production via negative feedback inhibition on the hypothalamus and the pituitary.

Due to its lipophilic nature, 90% of the total circulating cortisol is bound to a protein carrier (80% to cortisol-binding globulin (CBG) and 10% to albumin), the other 10% is unbound in the blood and therefore biologically active. Cortisol plays an important role in the stress response and contributes to the provision of energy by increasing catabolism and delaying anabolism. Other important systems requiring adequate levels of cortisol for normal functioning include the immune system, cardiovascular system, fluid and electrolyte homeostasis. Cortisol exerts its effects by binding to the intracellular glucocorticoid receptor (GR). Unbound, inactive GR resides in the cytoplasm as part of a multimeric complex with one or more heat shock proteins (hsp90). Upon cortisol binding, the multimeric complex dissolves and GR is transported to the nucleus where it regulates gene expression. Several receptor isoforms and subtypes with unique functional profiles are derived from a single gene (NR3C1) by alternative splicing and posttranslational modifications.

### The adrenal axis during critical illness

**Figure 3:**
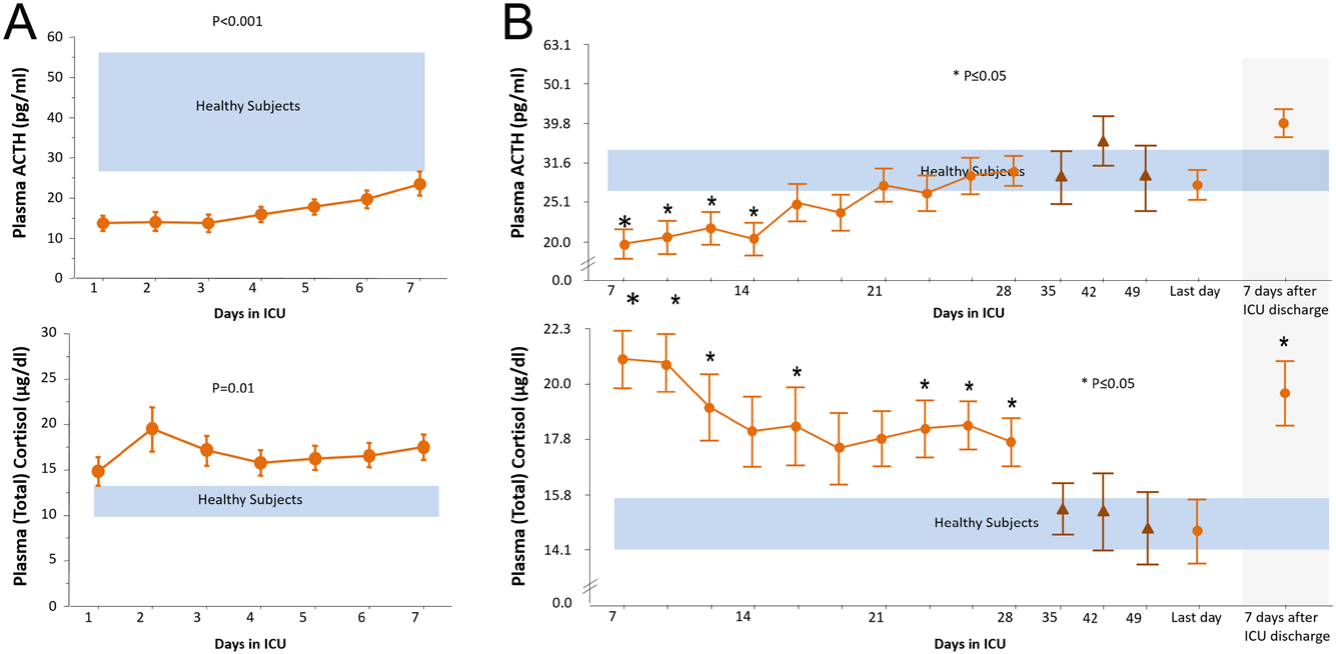
Changes in the ACTH and cortisol during critical illness. (A) The ACTH–cortisol dissociation, that is high levels of cortisol and low levels of ACTH, during the first week of ICU stay. Adapted, with permission, from (47). (B) The time course of HPA axis alteration beyond the first week of critical illness. The ACTH–cortisol dissociation appears to continue throughout the first month of ICU stay. In ‘very long stay’ patients (>4 weeks in ICU) cortisol and ACTH plasma concentrations gradually return to normal levels, despite their severity of illness. The blue areas indicate the range of healthy individuals. Adapted by permission from Springer Nature: *Intensive Care Medicine*; Adrenocortical function during prolonged critical illness and beyond: a prospective observational study, Peeters B, Meersseman P, Vander Perre S, Wouters PJ, Vanmarcke D, Debaveye Y, Billen J, Vermeersch P, Langouche L & Van den Berghe G; copyright 2018 (46).

A hallmark of critical illness, irrespective of the causal event, is the increase in plasma concentrations of the stress hormone cortisol. It was long assumed that the sustained several-fold rise in cortisol levels following a deleterious insult was caused by ongoing central, ACTH-driven adrenal cortisol synthesis and secretion (44). However, this concept now no longer stands as several studies have reported low rather than high ACTH plasma concentrations already from admission to the ICU onward (Fig. 3) (45). Moreover the diurnal rhythm of ACTH and cortisol seemed to be lost (45). In a recent prospective observational study, it was demonstrated that this ACTH–cortisol dissociation was present in all ICU patients, with or without sepsis/septic shock and survivors and non-survivors alike, and that it protracted throughout ICU stay. However, after a prolonged ICU stay of more than 4 weeks, cortisol levels decreased to normal levels, without a concomitant rise in ACTH (46).

As ACTH levels are low, the increase in systemic cortisol availability has to be brought about by non-ACTH-driven mechanisms. By using stable isotopes, elevated cortisol levels could indeed be attributed to suppressed cortisol metabolism, and, only in patients suffering from hyperinflammation, to a moderately increased cortisol production (47). The attenuated cortisol metabolism seems to be brought by reduced hepatic expression and activity of cortisol-metabolizing enzymes 5α- and 5β-reductase and renal 11β-hydroxysteroid dehydrogenase-2 (11βHSD2) (47). Cortisol-binding proteins CBG and albumin, also suppressed throughout ICU stay, further increase circulating levels of free cortisol and thus systemic cortisol availability (46, 47). A recent experimental mouse study documented a key role of reduced signaling of the hepatic GR in both suppression of cortisol metabolism and lowering levels of cortisol-binding proteins and thus attributing to hypercortisolemia (48).

The local tissue effects of highly elevated free cortisol levels during critical illness, such as modulation of the immune response, enhancement of hemodynamics and provision of energy, could also be affected by altered GR expression in various target organs. In analogy with the observed reduction of hepatic GR expression, the activity and expression of the active GR isoform (GRα) in target cells found in immune tissue seems to be reduced, whereas the negative GR isoform (GRβ) was transiently increased (49). This so-called ‘glucocorticoid resistance’, if present in other tissues such as the vasculature, and seemingly associated with disease severity, would pose a problem during critical illness as it would counteract any potential beneficial effects of cortisol (50). However, further research on tissue-specific changes of GR expression during critical illness is required.

Altogether, depressed ACTH-dependent secretion altered cortisol metabolism, and tissue-specific glucocorticoid resistance could lead to a state during critical illness in which the systemic cortisol availability could be insufficient for survival and recovery. Initially referred to as ‘relative adrenal insufficiency’, this state is nowadays labeled ‘critical illness-related corticosteroid insufficiency’ (CIRCI). In contrast to the occurrence of absolute adrenal failure in critically ill patients, neither a clear definition nor reliable diagnostic criteria nor an irrefutable treatment of CIRCI exist, as illustrated by the lack of consensus in recent guidelines (51, 52, 53). However, recent research revealed that cosyntropin stimulation tests are confounded by the increased cortisol distribution volume during critical illness. Indeed, low total cortisol responses to cosyntropin during critical illness rather reflect the increased cortisol distribution volume, given the low plasma binding, and can thus not provide reliable information on the functional status of the adrenal cortex (52). Controversy regarding the treatment of CIRCI and also regarding the overall use of glucocorticoids in septic shock, was further evoked by the recent publication of two large RCTs: the APROCCHSS trial and the ADRENAL trial (54, 55). In the APROCCHSS trial, irrespective of CIRCI as defined by the response to cosyntropin, 90-day all-cause mortality was lower among patients receiving hydrocortisone-plus-fludrocortisone compared to placebo, whereas this outcome did not significantly differ in the ADRENAL trial (54, 55). Although differences in inclusion and exclusion criteria, studying different stages of sepsis, as well as in the therapeutic agent, dosage and posology used, could partly explain the discordant findings, the studies contribute to the long-lasting controversy of adjunctive glucocorticoid therapy in critical illness (56).

The recent findings that low cortisol plasma-binding proteins and suppressed cortisol breakdown determine the systemic free cortisol availability during the first 4 weeks of critical illness, but that cortisol plasma concentrations return to normal levels beyond that timeframe, despite high severity of illness, suggests that especially these prolonged stayers might require treatment (46). Indeed, the persistently low levels of ACTH during a prolonged ICU stay could eventually lead to adrenal atrophy. This hypothesis is supported by the ten-fold higher prevalence of absolute adrenal insufficiency in ICU long-stayers (>14 days) compared to shorter-stayers (57). Also, only for patients with a prolonged ICU stay, adrenal atrophy and suppressed ACTH-regulated gene expression was documented postmortem (58). Whether these long stay patients would benefit from treatment with ACTH (or CRH) infusion, over exogenous glucocorticoids, in analogy with what has been described for the thyroidal axis should be investigated in future studies.

### The lactotropic axis during health

In non-pregnant humans, lactotropes secrete a burst of prolactin (PRL) every 2–3 h, varying in amplitude throughout the day (59). Unlike for other hypophyseal hormones, lactotropes show a high grade of spontaneous intrinsic activity. Indeed, when hypothalamic control is withheld, an unrestrained secretion of PRL is observed (60). In the normal physiological state, however, this intrinsic high-secretory tone is suppressed by dopamine (DA). Far less potent than DA, several other hormones such as TRH, oxytocin and vasoactive intestinal peptide (VIP) are known stimulators of PRL synthesis and secretion. Besides its important reproductive role by stimulating lactation and maternal behavior, PRL also affects several other functions such as maintenance of immune competence of lymphocytes and liver growth and is crucial for survival (61).

### The lactotropic axis during critical illness

In patients with sepsis or septic shock a clear rise in PRL levels is seen in the first days after the occurrence of a stressful life-threatening event (62). Interestingly, in one study of critically ill patients without sepsis or shock, prolactin levels on admission did not differ with those of matched healthy controls (63). When critical illness is prolonged, PRL levels start to decrease and eventually becomes suppressed. The mechanism behind this observation is not clear; however, a role of endogenous and exogenous DA has been suggested (33).

### The gonadal axis during health

Like most other hypothalamic hormones, GnRH is discharged in pulses into the hypophyseal portal system. At the level of the pituitary, GnRH stimulates the gonadotropes to synthesize and release stored luteinizing hormone (LH) and follicle-stimulating hormone (FSH). GnRH pulses are typically seen every 60–90 min and seem to be crucial for normal reproductive functioning (64). Whereas fluctuating gonadotropin-releasing hormone (GnRH) serum concentrations highly correlate with LH serum concentrations, FSH peaks are obscured and fluctuate less, partially caused by the longer biological half-life of FSH (65). LH stimulates steroidogenesis, in men predominantly testosterone by the testicular Leydig cells and in women primarily estrogens by the ovaries. A distinct function of LH in women is induction of ovulation and luteinization, both initiated by the surge in LH in the middle of the menstrual cycle. FSH stimulates folliculogenesis in the ovaries and spermatogenesis by the testicular Sertoli cells. Various fast and slow feedback loops both negative and positive, often intercrossing with other hormonal axes, reflect the complexity of the gonadal axis.

### The gonadal axis during critical illness

Gonadal steroid levels decrease after the onset of severe illness. In men, testosterone levels drop in face of apparently normal or even high LH levels (66). Cytokines that can reduce Leydig cell function and increase peripheral aromatization of androgens seem to mediate this effect (67). When recovery is not initiated and critical illness is prolonged, testosterone levels further decrease and may become unmeasurably low (68). The pulsatile pattern and amplitudes of LH fall and eventually lead to severe hypogonadotropism (69).

Levels of female gonadal hormones, estrogen and progesterone, are decreased in women who experience abnormal levels of stress, such as women with anxiety disorders, female athletes and women with various chronic diseases. This estrogen and progesterone deficiency is presumed to be the result of a combination of a central inhibition of the gonadal axis, through increased portal levels of CRH, because of an activated HPA axis and inhibition at the level of the hypothalamus, pituitary and ovaries exerted by the increase in systemic cortisol availability (70). However, most studies supporting this hypothesis arise from research studying minor physical and psychological stress. Furthermore, as described earlier, the central component of the HPA axis is rather suppressed instead of activated during prolonged critical illness. Importantly, most female ICU patients are post-menopausal and have an altered hypothalamic–pituitary–gonadal axis homeostasis. In these patients a paradoxically rise in estrogen levels is seen, presumably caused by an increase in peripheral aromatase activity (71).

In the early 2000s, a growing body of evidence suggested that the administration of the anabolic synthetic androgenic steroid oxandrolone in patients suffering from severe burns was associated with shorter hospital stay (72). Furthermore, in pediatric burn patients the use of oxandrolone was associated with fewer long-term catabolic complications such as burn-induced growth arrest (73). Despite the promising results of supplementation of estrogens in several animal models of critical illness, including traumatic brain injury and hemorrhagic shock, well-designed human studies are lacking (74, 75).

Studies on the use of androgens in prolonged critical illness failed to demonstrate any conclusive clinical benefit. Exogenous pulsatile GnRH administration, given together with GHRP2 and TRH infusion, induced an anabolic response, but research focusing on the potential clinical outcome benefit is still lacking (76).

## Are the neuroendocrine changes illness- or fasting-induced in the acute phase?

### Cytokines

At the onset of critical illness, cells from the innate immune system are activated by binding of pathogen-associated and damage-associated molecular patterns on the pattern recognition receptors. Following such activation, most leukocytes will release a number of small protein mediators, such as cytokines, which play an important role not only in the proinflammatory, but also in the anti-inflammatory response to critical illness. Circulating cytokines are able to induce or repress the production of other cytokines, creating a complex interplay, also called ‘cytokine networks’, with an important role in the pathogenesis of critical illness (77, 78).

Cytokines have been proposed to play an important role in the increase in circulating levels of pituitary hormones GH and PRL, the swift, transient rise in TSH and the decrease in plasma concentrations of ACTH, possibly through mediating an inflammatory response in the pituitary gland (79, 80). Moreover, at the level of the hypothalamus, cytokines as stimulators of local nitric oxygen (NO) secretion were shown to be able to induce apoptosis of hypothalamic neurons and glial cells (81). In a small post mortem study, reduced pituitary ACTH levels without a concomitant rise in hypothalamic CRH or vasopressin expression with increased iNOS expression have been reported in patients who died after septic shock (82). In contrast, the observation that in patients who recovered from critical illness ACTH and cortisol levels rose to supra-normal levels 1 week after ICU discharge (46) and suppressed ACTH responses to CRH infusion in the prolonged but not acute phase of critical illness (83), argues against a severely damaged hypothalamus and pituitary in critically ill patients. Moreover, in an experimental study, neutralization of TNF did not influence circulating levels of thyroid hormone or TSH (84). These mechanisms are distinct from the anatomical damage to the hypothalamus and/or the pituitary present in some patients suffering from traumatic brain injury (85).

### Central feedback inhibition

Besides cytokines, the increase in circulating GH can also be explained by a decrease in feedback due to low effector hormone IGF-I (52). Also, the observed increase in peripheral PRL levels could be mediated by altered stimulating and inhibiting effects of dopamine, oxytocin and VIP (80). High circulating levels of total and free cortisol on the other hand could exert a strong inhibitory effect on the hypothalamus and pituitary through negative feedback (83). This hypothesis is supported by the observation that ACTH levels fully recover or even become elevated after ICU discharge (46). Among other potential contributors to a centrally suppressed HPA axis are bile acids, as these are elevated during critical illness and have been observed to suppress the HPA axis in patients with and animal models of cholestasis (86). Altogether, pituitary hormone synthesis and release is highly influenced by feedback of the effector hormones, which may take place between the different pituitary cell types (87).

### Drugs

Patients admitted to the ICU often receive multiple drugs to support vital organ function. Several drugs frequently used at the modern ICU are well-known inhibitors of the adrenal axis in a dose-dependent manner and may possibly contribute to decreased ACTH production and secretion at the level of the pituitary (88). However, nowadays obsolete in most ICUs, nearly half a century ago intravenous infusion of dopamine was the first choice as inotropic agent, as historic studies showed a mortality benefit in critically ill patients with septic and cardiogenic shock with renal- and splanchic-sparing effects (89). However, two decades ago, it became clear that infusion with dopamine, in analogy with increased endogenous dopamine levels, aggravates suppression of circulating pituitary-dependent hormones, TSH, PRL and LH in the acute phase of critical illness and GH in the chronic phase (90).

### Nutritional signals

A third mechanism that may drive the alterations in pituitary and peripheral hormones is the lack of full (enteral) nutrition in the acute phase of critical illness. As critically ill patients are often unable to eat by mouth, nutritional support has to be initiated by the caregiving physician. However, nutrition guidelines recommend the early initiation of enteral feeding (EN) in most patients (91, 92), recent RCTs have questioned the ideal time to start parenteral nutrition (PN) if enteral feeding fails to meet the prespecified nutritional target (30, 31, 93, 94). Interestingly, most neuroendocrine changes in the acute phase of critical illness resemble those during fasting in healthy individuals: an increase in GH in face of low levels of IGF-I, a decrease in T3 with concomitant rise in rT3 despite relatively normal levels of TSH and T4 (NTI), a rise in systemic cortisol availability and a decrease in gonadal steroid hormones (29, 95). In this view, it is likely that the neuroendocrine adaptations in the acute phase of critical illness are beneficial or at least evolutionary selected and may enhance the chances of survival and recovery. Furthermore, accepting an early macronutrient deficit in critically ill patients by withholding PN in the (hyper)acute phase of critical illness was found to aggravate NTI in the late PN cohort, which statistically explained at least part of the outcome benefit of not feeding early (37, 96).

## A uniform central suppression in the chronic phase of critical illness

**Figure 4:**
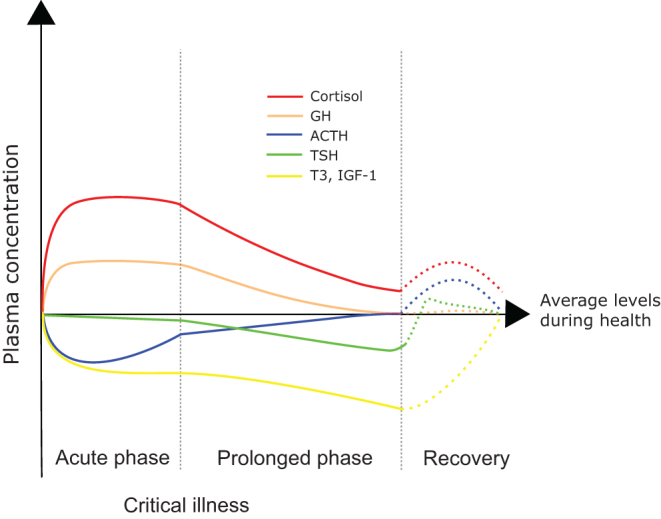
Simplified cartoon depicting the biphasic neuroendocrine response to critical illness. Trends in plasma concentrations of the most important pituitary and peripheral hormones during critical illness are rendered over time and compared to the physiological ranges in healthy individuals (black line). A rise in growth hormone levels is seen in the first hours after the onset of critical illness (orange line). This rise in GH coincides with a decrease in IGF-I (yellow line). During the chronic phase of critical illness, IGF-I further decreases and GH plasma concentration start to normalize. Thyroid hormone T3 levels rapidly decreases after the onset of critical illness with a further decline during the prolonged phase of critical illness (yellow line). It is currently unclear when the plasma levels of both IGF-1 and T3 fully normalize (dotted yellow line). Although TSH levels (green line) are not significantly altered during the first hours and days of critical illness, plasma concentration decreases when chronic critical illness sets in. When recovery is commenced, TSH transiently rise to supra-normal concentration before returning to physiological levels. Cortisol levels (red line) rise after a severe insult. High cortisol levels plateau in the first week of critical illness. When critical illness is prolonged, cortisol levels start to decrease. ACTH levels are rapidly reduced in acute critical illness but start to normalize after several days of critical illness. During the recovery phase, a rise in plasma concentrations of both ACTH and cortisol is seen (dotted blue line and dotted red line); however, when this rise is dampened and the circulating levels of ACTH and cortisol start to normalize is not clear.

The chronic phase of critical illness is hallmarked by a uniform suppression of all neuroendocrine axes (Fig. 4). Some of the proposed drivers of the neuroendocrine changes in the acute phase of critical illness are unlikely to play a prominent role in the chronic phase. Cytokines could be involved, although their levels substantially decline during the time course of severe illness (97). Also nutritional signals are different compared to patients in the acute phase of critical illness as most patients are now fully fed, either by enteral feeding, parenteral feeding or a combination of both (98).

The most plausible mechanism behind this uniform pituitary suppression is probably the decrease in hypothalamic, mostly activating, hypophysiotropic hormones. Indeed, a post mortem analysis of hypothalamic tissue demonstrated markedly reduced TRH gene expression in patients who died after severe illness compared to healthy patients who succumbed from a sudden lethal insult (99). Furthermore, ongoing hypercortisolemia and high levels of endogenous dopamine may enhance negative feedback and therefore contribute to decreased pituitary hormone production and secretion (100).

Interestingly, hypothalamic type 2 deiodinase (D2) and local expression of thyroid hormone transporters are increased during critical illness and could theoretically increase local T3 availability and therefore alter the set point for feedback inhibition (101). Whether such alteration in set point for feedback is present in other pituitary cell types is unknown.

## Conclusion

During critical illness, the neuroendocrine axes are altered in a biphasic manner (Fig. 4). Neuroendocrine changes during the acute phase of critical illness resemble, at least partially, a fasted state and seem to be evolutionary selected and likely beneficial for survival. Therefore, treatment of altered hormone levels in this phase of critical illness might not be indicated. When patients fail to recover sufficiently, central and peripheral hormone levels further alter. These profound alterations no longer represent a natural fasting state and could be interpreted as maladaptive and may hamper recovery. Although treatment with exogenous peripherally active hormones in this more chronic phase of critical illness seems a reasonable option, experimental studies in the past have highlighted difficulties with optimal dosing and posology, sometimes even causing harm. A more physiological solution would be the use of central releasing factors instead of peripheral hormones, allowing a normal feedback regulation which would avoid toxic levels of peripherally active hormones. Further intervention studies are needed to investigate the future role of treatment with releasing factors in chronic critical illness.

## Declaration of interest

The authors declare that there is no conflict of interest that could be perceived as prejudicing the impartiality of this review.

## Funding

This work was supported by the Research Foundation Flanders (FWO) grant G091918N to G V d B, the European Research Council Advanced Grant (AdvG-2017-785806 to G V d B) from European Union’s Horizon 2020 research and innovation programme and the Methusalem programme of the Flemish Government (METH/14/06 to G V d B and L L via the KU Leuven).

## References

[1] Nelson JE Cox CE Hope AA Carson SS Chronic critical illness. American Journal of Respiratory and Critical Care Medicine 2010 182 446–454. (10.1164/rccm.201002-0210CI) 20448093 PMC2937238

[2] Iwashyna TJ Hodgson CL Pilcher D Bailey M van Lint A Chavan S Bellomo R Timing of onset and burden of persistent critical illness in Australia and New Zealand: a retrospective, population-based, observational study. Lancet: Respiratory Medicine 2016 4 566–573. (10.1016/S2213-2600(16)30098-4) 27155770

[3] Vanhorebeek I Langouche L Van den Berghe G Endocrine aspects of acute and prolonged critical illness. Nature Clinical Practice: Endocrinology and Metabolism 2006 2 20–31. (10.1038/ncpendmet0071) 16932250

[4] Van den Berghe G de Zegher F Bouillon R Clinical review 95: acute and prolonged critical illness as different neuroendocrine paradigms. Journal of Clinical Endocrinology and Metabolism 1998 83 1827–1834. (10.1210/jcem.83.6.4763) 9626104

[5] Gheorghita V Barbu AE Gheorghiu ML Caruntu FA Endocrine dysfunction in sepsis: a beneficial or deleterious host response? Germs 2015 5 17–25. (10.11599/germs.2015.1067) 25763364 PMC4350863

[6] Maddens M Sowers J Catecholamines in critical care. Critical Care Clinics 1987 3 871–882. (10.1016/S0749-0704(18)30524-4) 3332227

[7] Thiessen SE Van den Berghe G Vanhorebeek I Mitochondrial and endoplasmic reticulum dysfunction and related defense mechanisms in critical illness-induced multiple organ failure. Biochimica et Biophysica Acta: Molecular Basis of Disease 2017 1863 2534–2545. (10.1016/j.bbadis.2017.02.015) 28219766

[8] Singer M Critical illness and flat batteries. Critical Care 2017 21 309 (10.1186/s13054-017-1913-9) 29297363 PMC5751585

[9] Giustina A Veldhuis JD Pathophysiology of the neuroregulation of growth hormone secretion in experimental animals and the human. Endocrine Reviews 1998 19 717–797. (10.1210/edrv.19.6.0353) 9861545

[10] Takaya K Ariyasu H Kanamoto N Iwakura H Yoshimoto A Harada M Mori K Komatsu Y Usui T Shimatsu A, ***et al*** Ghrelin strongly stimulates growth hormone release in humans. Journal of Clinical Endocrinology and Metabolism 2000 85 4908–4911. (10.1210/jcem.85.12.7167) 11134161

[11] Tschop M Smiley DL Heiman ML Ghrelin induces adiposity in rodents. Nature 2000 407 908–913. (10.1038/35038090) 11057670

[12] Ross R Miell J Freeman E Jones J Matthews D Preece M Buchanan C Critically ill patients have high basal growth hormone levels with attenuated oscillatory activity associated with low levels of insulin-like growth factor-I. Clinical Endocrinology 1991 35 47–54. (10.1111/j.1365-2265.1991.tb03495.x) 1909610

[13] Teng Chung T Hinds CJ Treatment with GH and IGF-1 in critical illness. Critical Care Clinics 2006 22 29–40, vi (10.1016/j.ccc.2005.09.003) 16399018

[14] Timmins AC Cotterill AM Hughes SC Holly JM Ross RJ Blum W Hinds CJ Critical illness is associated with low circulating concentrations of insulin-like growth factors-I and -II, alterations in insulin-like growth factor binding proteins, and induction of an insulin-like growth factor binding protein 3 protease. Critical Care Medicine 1996 24 1460–1466. (10.1097/00003246-199609000-00006) 8797616

[15] Bentham J Rodriguez-Arnao J Ross RJ Acquired growth hormone resistance in patients with hypercatabolism. Hormone Research 1993 40 87–91. (10.1159/000183772) 7507879

[16] Van den Berghe G de Zegher F Veldhuis JD Wouters P Awouters M Verbruggen W Schetz M Verwaest C Lauwers P Bouillon R, ***et al*** The somatotropic axis in critical illness: effect of continuous growth hormone (GH)-releasing hormone and GH-releasing peptide-2 infusion. Journal of Clinical Endocrinology and Metabolism 1997 82 590–599. (10.1210/jcem.82.2.3736) 9024260

[17] Van den Berghe G Growth hormone secretagogues in critical illness. Hormone Research 1999 51 (Supplement 3) 21–28. (10.1159/000053158) 10592440

[18] Van den Berghe G de Zegher F Baxter RC Veldhuis JD Wouters P Schetz M Verwaest C Van der Vorst E Lauwers P Bouillon R, ***et al*** Neuroendocrinology of prolonged critical illness: effects of exogenous thyrotropin-releasing hormone and its combination with growth hormone secretagogues. Journal of Clinical Endocrinology and Metabolism 1998 83 309–319. (10.1210/jcem.83.2.4575) 9467533

[19] Hill NE Murphy KG Singer M Ghrelin, appetite and critical illness. Current Opinion in Critical Care 2012 18 199–205. (10.1097/MCC.0b013e3283514b01) 22322262

[20] Van den Berghe G Wouters P Weekers F Mohan S Baxter RC Veldhuis JD Bowers CY Bouillon R Reactivation of pituitary hormone release and metabolic improvement by infusion of growth hormone-releasing peptide and thyrotropin-releasing hormone in patients with protracted critical illness. Journal of Clinical Endocrinology and Metabolism 1999 84 1311–1323. (10.1210/jcem.84.4.5636) 10199772

[21] Takala J Ruokonen E Webster NR Nielsen MS Zandstra DF Vundelinckx G Hinds CJ Increased mortality associated with growth hormone treatment in critically ill adults. New England Journal of Medicine 1999 341 785–792. (10.1056/NEJM199909093411102) 10477776

[22] Van den Berghe G de Zegher F Bowers CY Wouters P Muller P Soetens F Vlasselaers D Schetz M Verwaest C Lauwers P, ***et al*** Pituitary responsiveness to GH-releasing hormone, GH-releasing peptide-2 and thyrotrophin-releasing hormone in critical illness. Clinical Endocrinology 1996 45 341–351. (10.1046/j.1365-2265.1996.00805.x) 8949573

[23] Zhou M Aziz M Ochani M Yang WL Sharma A Wang P The protective role of human ghrelin in sepsis: restoration of CD4 T cell proliferation. PLoS ONE 2018 13 e0201139 (10.1371/journal.pone.0201139) 30052667 PMC6063405

[24] Narula T deBoisblanc BP Ghrelin in critical illness. American Journal of Respiratory Cell and Molecular Biology 2015 53 437–442. (10.1165/rcmb.2014-0226TR) 26068568

[25] Samuels MH Veldhuis JD Henry P Ridgway EC Pathophysiology of pulsatile and copulsatile release of thyroid-stimulating hormone, luteinizing hormone, follicle-stimulating hormone, and alpha-subunit. Journal of Clinical Endocrinology and Metabolism 1990 71 425–432. (10.1210/jcem-71-2-425) 1696277

[26] Chopra IJ Huang TS Beredo A Solomon DH Chua GN Mead JF Evidence for an inhibitor of extrathyroidal conversion of thyroxine to 3,5,3′-triiodothyronine in sera of patients with nonthyroidal illnesses. Journal of Clinical Endocrinology and Metabolism 1985 60 666–672. (10.1210/jcem-60-4-666) 2857729

[27] Peeters RP Wouters PJ Kaptein E van Toor H Visser TJ Van den Berghe G Reduced activation and increased inactivation of thyroid hormone in tissues of critically ill patients. Journal of Clinical Endocrinology and Metabolism 2003 88 3202–3211. (10.1210/jc.2002-022013) 12843166

[28] Bartalena L Martino E Brandi LS Falcone M Pacchiarotti A Ricci C Bogazzi F Grasso L Mammoli C Pinchera A Lack of nocturnal serum thyrotropin surge after surgery. Journal of Clinical Endocrinology and Metabolism 1990 70 293–296. (10.1210/jcem-70-1-293) 2294138

[29] Boelen A Wiersinga WM Fliers E Fasting-induced changes in the hypothalamus-pituitary-thyroid axis. Thyroid 2008 18 123–129. (10.1089/thy.2007.0253) 18225975

[30] Casaer MP Mesotten D Hermans G Wouters PJ Schetz M Meyfroidt G Van Cromphaut S Ingels C Meersseman P Muller J, ***et al*** Early versus late parenteral nutrition in critically ill adults. New England Journal of Medicine 2011 365 506–517. (10.1056/NEJMoa1102662) 21714640

[31] Fivez T Kerklaan D Mesotten D Verbruggen S Wouters PJ Vanhorebeek I Debaveye Y Vlasselaers D Desmet L Casaer MP, ***et al*** Early versus late parenteral nutrition in critically ill children. New England Journal of Medicine 2016 374 1111–1122. (10.1056/NEJMoa1514762) 26975590

[32] van der Spek AH Jim KK Karaczyn A van Beeren HC Ackermans MT Darras VM Vandenbroucke-Grauls CMJE Hernandez A Brouwer MC Fliers E, ***et al*** The thyroid hormone inactivating type 3 deiodinase is essential for optimal neutrophil function: observations from three species. Endocrinology 2018 159 826–835. (10.1210/en.2017-00666) 29186449 PMC5774253

[33] Van den Berghe G de Zegher F Veldhuis JD Wouters P Gouwy S Stockman W Weekers F Schetz M Lauwers P Bouillon R, ***et al*** Thyrotrophin and prolactin release in prolonged critical illness: dynamics of spontaneous secretion and effects of growth hormone-secretagogues. Clinical Endocrinology 1997 47 599–612. (10.1046/j.1365-2265.1997.3371118.x) 9425400

[34] Mebis L Paletta D Debaveye Y Ellger B Langouche L D'Hoore A Darras VM Visser TJ Van den Berghe G Expression of thyroid hormone transporters during critical illness. European Journal of Endocrinology 2009 161 243–250. (10.1530/EJE-09-0290) 19439506

[35] Mebis L Langouche L Visser TJ Van den Berghe G The type II iodothyronine deiodinase is up-regulated in skeletal muscle during prolonged critical illness. Journal of Clinical Endocrinology and Metabolism 2007 92 3330–3333. (10.1210/jc.2007-0510) 17504898

[36] Rothwell PM Udwadia ZF Lawler PG Thyrotropin concentration predicts outcome in critical illness. Anaesthesia 1993 48 373–376. (10.1111/j.1365-2044.1993.tb07006.x) 8317642

[37] Langouche L Vander Perre S Marques M Boelen A Wouters PJ Casaer MP Van den Berghe G Impact of early nutrient restriction during critical illness on the nonthyroidal illness syndrome and its relation with outcome: a randomized, controlled clinical study. Journal of Clinical Endocrinology and Metabolism 2013 98 1006–1013. (10.1210/jc.2012-2809) 23348400

[38] Fliers E Bianco AC Langouche L Boelen A Thyroid function in critically ill patients. Lancet: Diabetes and Endocrinology 2015 3 816–825. (10.1016/S2213-8587(15)00225-9) 26071885 PMC4979220

[39] Debaveye Y Ellger B Mebis L Darras VM Van den Berghe G Regulation of tissue iodothyronine deiodinase activity in a model of prolonged critical illness. Thyroid 2008 18 551–560. (10.1089/thy.2007.0287) 18466079

[40] Debaveye Y Ellger B Mebis L Van Herck E Coopmans W Darras V Van den Berghe G Tissue deiodinase activity during prolonged critical illness: effects of exogenous thyrotropin-releasing hormone and its combination with growth hormone-releasing peptide-2. Endocrinology 2005 146 5604–5611. (10.1210/en.2005-0963) 16150898

[41] Debaveye Y Ellger B Mebis L Visser TJ Darras VM Van den Berghe G Effects of substitution and high-dose thyroid hormone therapy on deiodination, sulfoconjugation, and tissue thyroid hormone levels in prolonged critically ill rabbits. Endocrinology 2008 149 4218–4228. (10.1210/en.2007-1566) 18450965 PMC2488214

[42] Gambacciani M Liu JH Swartz WH Tueros VS Rasmussen DD Yen SS Intrinsic pulsatility of ACTH release from the human pituitary in vitro. Clinical Endocrinology 1987 26 557–563. (10.1111/j.1365-2265.1987.tb00810.x) 2822296

[43] Novoselova T King P Guasti L Metherell LA Clark AJL Chan LF ACTH signalling and adrenal development: lessons from mouse models. Endocrine Connections 2019 8 R122–R130. (10.1530/EC-19-0190) PMC6652236 31189126

[44] Lamberts SW Bruining HA de Jong FH Corticosteroid therapy in severe illness. New England Journal of Medicine 1997 337 1285–1292. (10.1056/NEJM199710303371807) 9345079

[45] Peeters B Langouche L Van den Berghe G Adrenocortical stress response during the course of critical illness. Comprehensive Physiology 2017 8 283–298. (10.1002/cphy.c170022) 29357129

[46] Peeters B Meersseman P Vander Perre S Wouters PJ Vanmarcke D Debaveye Y Billen J Vermeersch P Langouche L Van den Berghe G Adrenocortical function during prolonged critical illness and beyond: a prospective observational study. Intensive Care Medicine 2018 44 1720–1729. (10.1007/s00134-018-5366-7) 30215187 PMC6182356

[47] Boonen E Vervenne H Meersseman P Andrew R Mortier L Declercq PE Vanwijngaerden YM Spriet I Wouters PJ Vander Perre S, ***et al*** Reduced cortisol metabolism during critical illness. New England Journal of Medicine 2013 368 1477–1488. (10.1056/NEJMoa1214969) 23506003 PMC4413428

[48] Jenniskens M Weckx R Dufour T Vander Perre S Pauwels L Derde S Teblick A Guiza F Van den Berghe G Langouche L The hepatic glucocorticoid receptor is crucial for cortisol homeostasis and sepsis survival in humans and male mice. Endocrinology 2018 159 2790–2802. (10.1210/en.2018-00344) 29788135

[49] Ledderose C Mohnle P Limbeck E Schutz S Weis F Rink J Briegel J Kreth S Corticosteroid resistance in sepsis is influenced by microRNA-124-induced downregulation of glucocorticoid receptor-alpha. Critical Care Medicine 2012 40 2745–2753. (10.1097/CCM.0b013e31825b8ebc) 22846781

[50] Cohen J Pretorius CJ Ungerer JP Cardinal J Blumenthal A Presneill J Gatica-Andrades M Jarrett P Lassig-Smith M Stuart J, ***et al*** Glucocorticoid sensitivity is highly variable in critically ill patients with septic shock and is associated with disease severity. Critical Care Medicine 2016 44 1034–1041. (10.1097/CCM.0000000000001633) 26963327

[51] Boonen E Van den Berghe G MECHANISMS IN ENDOCRINOLOGY: New concepts to further unravel adrenal insufficiency during critical illness. European Journal of Endocrinology 2016 175 R1–R9. (10.1530/EJE-15-1098) 26811405

[52] Téblick A Peeters B Langouche L Van den Berghe G Adrenal function and dysfunction in critically ill patients. Nature Reviews: Endocrinology 2019 15 417–427. (10.1038/s41574-019-0185-7) 30850749

[53] Annane D Pastores SM Rochwerg B Arlt W Balk RA Beishuizen A Briegel J Carcillo J Christ-Crain M Cooper MS, ***et al*** Guidelines for the diagnosis and management of critical illness-related corticosteroid insufficiency (CIRCI) in critically ill patients (Part I): Society of Critical Care Medicine (SCCM) and European Society of Intensive Care Medicine (ESICM) 2017. Intensive Care Medicine 2017 43 1751–1763. (10.1007/s00134-017-4919-5) 28940011

[54] Venkatesh B Finfer S Cohen J Rajbhandari D Arabi Y Bellomo R Billot L Correa M Glass P Harward M, ***et al*** Adjunctive glucocorticoid therapy in patients with septic shock. New England Journal of Medicine 2018 378 797–808. (10.1056/NEJMoa1705835) 29347874

[55] Annane D Renault A Brun-Buisson C Megarbane B Quenot JP Siami S Cariou A Forceville X Schwebel C Martin C, ***et al*** Hydrocortisone plus fludrocortisone for adults with septic shock. New England Journal of Medicine 2018 378 809–818. (10.1056/NEJMoa1705716) 29490185

[56] Marik PE The role of glucocorticoids as adjunctive treatment for sepsis in the modern era. Lancet: Respiratory Medicine 2018 6 793–800. (10.1016/S2213-2600(18)30265-0) 30006071

[57] Barquist E Kirton O Adrenal insufficiency in the surgical intensive care unit patient. Journal of Trauma 1997 42 27–31. (10.1097/00005373-199701000-00006) 9003254

[58] Boonen E Langouche L Janssens T Meersseman P Vervenne H De Samblanx E Pironet Z Van Dyck L Vander Perre S Derese I, ***et al*** Impact of duration of critical illness on the adrenal glands of human intensive care patients. Journal of Clinical Endocrinology and Metabolism 2014 99 4214–4222. (10.1210/jc.2014-2429) 25062464

[59] Sassin JF Frantz AG Kapen S Weitzman ED The nocturnal rise of human prolactin is dependent on sleep. Journal of Clinical Endocrinology and Metabolism 1973 37 436–440. (10.1210/jcem-37-3-436) 4361974

[60] Meites J Neuroendocrine control of prolactin in experimental animals. Clinical Endocrinology 1977 6 (Supplement 1) 9S–18S. (10.1111/j.1365-2265.1977.tb03334.x) 30556

[61] Bole-Feysot C Goffin V Edery M Binart N Kelly PA Prolactin (PRL) and its receptor: actions, signal transduction pathways and phenotypes observed in PRL receptor knockout mice. Endocrine Reviews 1998 19 225–268. (10.1210/edrv.19.3.0334) 9626554

[62] Nguyen DN Huyghens L Schiettecatte J Smitz J Vincent JL High prolactin levels are associated with more delirium in septic patients. Journal of Critical Care 2016 33 56–61. (10.1016/j.jcrc.2015.12.021) 26852394

[63] Mpouzika MD Papathanassoglou ED Giannakopoulou M Bozas E Middleton N Boti S Patiraki EI Karabinis A Altered serum stress neuropeptide levels in critically ill individuals and associations with lymphocyte populations. Neuropeptides 2013 47 25–36. (10.1016/j.npep.2012.07.007) 22981820

[64] Belchetz PE Plant TM Nakai Y Keogh EJ Knobil E Hypophysial responses to continuous and intermittent delivery of hypopthalamic gonadotropin-releasing hormone. Science 1978 202 631–633. (10.1126/science.100883) 100883

[65] Clarke IJ Two decades of measuring GnRH secretion. Reproduction Supplement 2002 59 1–13. 12698969

[66] Spratt DI Cox P Orav J Moloney J Bigos T Reproductive axis suppression in acute illness is related to disease severity. Journal of Clinical Endocrinology and Metabolism 1993 76 1548–1554. (10.1210/jcem.76.6.8501163) 8501163

[67] Eachempati SR Hydo L Barie PS Gender-based differences in outcome in patients with sepsis. Archives of Surgery 1999 134 1342–1347. (10.1001/archsurg.134.12.1342) 10593332

[68] Almoosa KF Gupta A Pedroza C Watts NB Low testosterone levels are frequent in patients with acute respiratory failure and are associated with poor outcomes. Endocrine Practice 2014 20 1057–1063. (10.4158/EP14003.OR) 24936547

[69] Woolf PD Hamill RW McDonald JV Lee LA Kelly M Transient hypogonadotropic hypogonadism caused by critical illness. Journal of Clinical Endocrinology and Metabolism 1985 60 444–450. (10.1210/jcem-60-3-444) 3919042

[70] Kalantaridou SN Makrigiannakis A Zoumakis E Chrousos GP Stress and the female reproductive system. Journal of Reproductive Immunology 2004 62 61–68. (10.1016/j.jri.2003.09.004) 15288182

[71] Raj MN Suresh V Mukka A Reddy A Sachan A Mohan A Vengamma B Rao PV Evaluation of activity of hypothalamo-pituitary-gonadal axis in postmenopausal women suffering from severe acute illness. Indian Journal of Medical Research 2016 143 66–71. (10.4103/0971-5916.178596) 26997016 PMC4822371

[72] Wolf SE Edelman LS Kemalyan N Donison L Cross J Underwood M Spence RJ Noppenberger D Palmieri TL Greenhalgh DG, ***et al*** Effects of oxandrolone on outcome measures in the severely burned: a multicenter prospective randomized double-blind trial. Journal of Burn Care and Research 2006 27 131–139; discussion 140–131. (10.1097/01.BCR.0000202620.55751.4F) 16566555

[73] Herndon DN Voigt CD Capek KD Wurzer P Guillory A Kline A Andersen CR Klein GL Tompkins RG Suman OE, ***et al*** Reversal of growth arrest With the combined administration of oxandrolone and propranolol in severely burned children. Annals of Surgery 2016 264 421–428. (10.1097/SLA.0000000000001844) 27433905 PMC5167626

[74] Wigginton JG Pepe PE Idris AH Rationale for routine and immediate administration of intravenous estrogen for all critically ill and injured patients. Critical Care Medicine 2010 38 S620–S629. (10.1097/CCM.0b013e3181f243a9) 21164406

[75] Weniger M Angele MK Chaudry IH The role and use of estrogens following trauma. Shock 2016 46 4–11. (10.1097/SHK.0000000000000670) 27380534

[76] Van den Berghe G Baxter RC Weekers F Wouters P Bowers CY Iranmanesh A Veldhuis JD Bouillon R The combined administration of GH-releasing peptide-2 (GHRP-2), TRH and GnRH to men with prolonged critical illness evokes superior endocrine and metabolic effects compared to treatment with GHRP-2 alone. Clinical Endocrinology 2002 56 655–669. (10.1046/j.1365-2265.2002.01255.x) 12030918

[77] Matsumoto H Ogura H Shimizu K Ikeda M Hirose T Matsuura H Kang S Takahashi K Tanaka T Shimazu T The clinical importance of a cytokine network in the acute phase of sepsis. Scientific Reports 2018 8 13995 (10.1038/s41598-018-32275-8) 30228372 PMC6143513

[78] Kox WJ Volk T Kox SN Volk HD Immunomodulatory therapies in sepsis. Intensive Care Medicine 2000 26 (Supplement 1) S124–S128. (10.1007/s001340051129) 10786969

[79] Annane D The role of ACTH and corticosteroids for sepsis and septic shock: an update. Frontiers in Endocrinology 2016 7 70 (10.3389/fendo.2016.00070) 27379022 PMC4913096

[80] Noel GL Suh HK Stone JG Frantz AG Human prolactin and growth hormone release during surgery and other conditions of stress. Journal of Clinical Endocrinology and Metabolism 1972 35 840–851. (10.1210/jcem-35-6-840) 4634485

[81] McCann SM Kimura M Karanth S Yu WH Mastronardi CA Rettori V The mechanism of action of cytokines to control the release of hypothalamic and pituitary hormones in infection. Annals of the New York Academy of Sciences 2000 917 4–18. (10.1111/j.1749-6632.2000.tb05368.x) 11268367

[82] Polito A Sonneville R Guidoux C Barrett L Viltart O Mattot V Siami S Lorin de la Grandmaison G Chretien F Singer M, ***et al*** Changes in CRH and ACTH synthesis during experimental and human septic shock. PLoS ONE 2011 6 e25905 (10.1371/journal.pone.0025905) 22073145 PMC3207830

[83] Peeters B Meersseman P Vander Perre S Wouters PJ Debaveye Y Langouche L Van den Berghe G ACTH and cortisol responses to CRH in acute, subacute, and prolonged critical illness: a randomized, double-blind, placebo-controlled, crossover cohort study. Intensive Care Medicine 2018 44 2048–2058. (10.1007/s00134-018-5427-y) 30374692 PMC6280831

[84] van der Poll T Endert E Coyle SM Agosti JM Lowry SF Neutralization of TNF does not influence endotoxininduced changes in thyroid hormone metabolism in humans. American Journal of Physiology 1999 276 R357–R362. (10.1152/ajpregu.1999.276.2.R357) 9950912

[85] Ntali G Tsagarakis S Traumatic brain injury induced neuroendocrine changes: acute hormonal changes of anterior pituitary function. Pituitary 2019 22 283–295. (10.1007/s11102-019-00944-0) 30746590

[86] McMillin M Frampton G Quinn M Divan A Grant S Patel N Newell-Rogers K DeMorrow S Suppression of the HPA axis during cholestasis can be attributed to hypothalamic bile acid signaling. Molecular Endocrinology 2015 29 1720–1730. (10.1210/me.2015-1087) 26431088 PMC4664228

[87] Vazquez-Borrego MC Gahete MD Martinez-Fuentes AJ Fuentes-Fayos AC Castano JP Kineman RD Luque RM Multiple signaling pathways convey central and peripheral signals to regulate pituitary function: lessons from human and non-human primate models. Molecular and Cellular Endocrinology 2018 463 4–22. (10.1016/j.mce.2017.12.007) 29253530

[88] Peeters B Guiza F Boonen E Meersseman P Langouche L Van den Berghe G Drug-induced HPA axis alterations during acute critical illness: a multivariable association study. Clinical Endocrinology 2017 86 26–36. (10.1111/cen.13155) 27422812

[89] Goldberg LI Dopamine – clinical uses of an endogenous catecholamine. New England Journal of Medicine 1974 291 707–710. (10.1056/NEJM197410032911405) 4604342

[90] Van den Berghe G de Zegher F Anterior pituitary function during critical illness and dopamine treatment. Critical Care Medicine 1996 24 1580–1590. (10.1097/00003246-199609000-00024) 8797634

[91] Singer P Blaser AR Berger MM Alhazzani W Calder PC Casaer MP Hiesmayr M Mayer K Montejo JC Pichard C, ***et al*** ESPEN guideline on clinical nutrition in the intensive care unit. Clinical Nutrition 2019 38 48–79. (10.1016/j.clnu.2018.08.037) 30348463

[92] McClave SA Taylor BE Martindale RG Warren MM Johnson DR Braunschweig C McCarthy MS Davanos E Rice TW Cresci GA, ***et al*** Guidelines for the provision and assessment of nutrition support therapy in the adult critically ill patient: Society of Critical Care Medicine (SCCM) and American Society for Parenteral and Enteral Nutrition (A.S.P.E.N.). Journal of Parenteral and Enteral Nutrition 2016 40 159–211. (10.1177/0148607115621863) 26773077

[93] Arabi YM Casaer MP Chapman M Heyland DK Ichai C Marik PE Martindale RG McClave SA Preiser JC Reignier J, ***et al*** The intensive care medicine research agenda in nutrition and metabolism. Intensive Care Medicine 2017 43 1239–1256. (10.1007/s00134-017-4711-6) 28374096 PMC5569654

[94] Van Dyck L Casaer MP Gunst J Autophagy and its implications against early full nutrition support in critical illness. Nutrition in Clinical Practice 2018 33 339–347. (10.1002/ncp.10084) 29665131

[95] Hartman ML Veldhuis JD Johnson ML Lee MM Alberti KG Samojlik E Thorner MO Augmented growth hormone (GH) secretory burst frequency and amplitude mediate enhanced GH secretion during a two-day fast in normal men. Journal of Clinical Endocrinology and Metabolism 1992 74 757–765. (10.1210/jcem.74.4.1548337) 1548337

[96] Jacobs A Derese I Vander Perre S van Puffelen E Verstraete S Pauwels L Verbruggen S Wouters P Langouche L Garcia Guerra G, ***et al*** Non-thyroidal illness syndrome in critically ill children: prognostic value and impact of nutritional management. Thyroid 2019 29 480–492. (10.1089/thy.2018.0420) 30760183 PMC6457888

[97] Damas P Reuter A Gysen P Demonty J Lamy M Franchimont P Tumor necrosis factor and interleukin-1 serum levels during severe sepsis in humans. Critical Care Medicine 1989 17 975–978. (10.1097/00003246-198910000-00001) 2791581

[98] Hollander JM Mechanick JI Nutrition support and the chronic critical illness syndrome. Nutrition in Clinical Practice 2006 21 587–604. (10.1177/0115426506021006587) 17119165

[99] Fliers E Guldenaar SE Wiersinga WM Swaab DF Decreased hypothalamic thyrotropin-releasing hormone gene expression in patients with nonthyroidal illness. Journal of Clinical Endocrinology and Metabolism 1997 82 4032–4036. (10.1210/jcem.82.12.4404) 9398708

[100] Van den Berghe G de Zegher F Lauwers P Dopamine and the sick euthyroid syndrome in critical illness. Clinical Endocrinology 1994 41 731–737. (10.1111/j.1365-2265.1994.tb02787.x) 7889608

[101] Mebis L Debaveye Y Ellger B Derde S Ververs EJ Langouche L Darras VM Fliers E Visser TJ Van den Berghe G Changes in the central component of the hypothalamus-pituitary-thyroid axis in a rabbit model of prolonged critical illness. Critical Care 2009 13 R147 (10.1186/cc8043) 19747372 PMC2784366

